# Tunable graphene-based mid-infrared plasmonic wide-angle narrowband perfect absorber

**DOI:** 10.1038/srep36651

**Published:** 2016-11-15

**Authors:** Hongju Li, Lingling Wang, Xiang Zhai

**Affiliations:** 1 School of Physics and Electronic, Hunan University, Changsha, 410082, China

## Abstract

In this paper, the periodic double-layer graphene ribbon arrays placed near a metallic ground plate coated by a dielectric layer are proposed and analyzed by the coupled-mode theory (CMT) to predict the perfect absorption response in the mid-infrared region. Numerical simulations of the finite-difference time-domain (FDTD) method confirm this effect and give the underlying physical origin. The anti-symmetric dipole-dipole coupling mode is supported by the double-layer graphene ribbons and acts as the electrical resonance to suppress the reflection, because of the impedance matching. The transmission from this system is restricted by the ultra-thick metallic ground plate. All incident electromagnetic energy is efficiently confined in the interlayer between graphene ribbons and the metallic plate, and the dramatic narrowband perfect absorption peak with the FWHM (full width at half maximums) of 300 nm hence is achieved. The spectral position of the absorption peak can be dynamically tuned by a small change in the chemical potential of graphene, in addition to varying geometrical parameters of the absorber. Meanwhile, this device exhibits good absorption stability over a wide angle range of incidence around ± 60° at least. Such absorber will benefit the fabrication of mid-infrared nano-photonic devices for optical filtering and storage.

Over the past decades, an extensive amount of research has been carried out on the metamaterials^1^,^2^ with unit cells on the sub-wavelength scale in virtue of the growing number of practical applications in the invisibility cloaking^3^, negative index of refraction^4^, and perfect lensing^5^. The metallic ribbons^6^, cut wire pairs^7^, and split ring resonators^8^ have been proposed to predict and enrich the metamaterial theory^9^,^10^. Various exotic features that are unavailable in nature have been achieved. Metamaterial perfect absorbers^11^,^12^, as an important branch of metamaterials, have attracted considerable attention currently, because the inevitable losses in metallic plasmonic nanostructures can be put to advantages for this research area. In order to realize near-unity absorption, the reflectance is suppressed by matching the effective impedance of the metamaterial to that of the incident medium. Simultaneously, the transmittance is eliminated via maximizing the metamaterial losses, for example, by introducing another metallic plate acting as the substrate^13^. So far, based on this mechanism, metamaterial near-unity absorbers with electrical resonators including metallic disks^14^, strips^15^,^16^, crosses^10^, and L-shaped structures^17^ have been designed and investigated comprehensively. By combining multiple resonators with different sizes together to form a super unit cell, the multi-channel or broadband perfect absorbers have also been achieved^18^,^19^,^20^,^21^. In addition, the method of the stack multilayer of resonators with different geometric parameters separated by dielectric layers has been used to realize the multi-channel or broadband absorption peaks^22^,^23^. However, in order to tune the spectral position of the absorption peak, only the passive method of changing structural parameters of metamaterials is utilized in the most cases. In the practical applications, the method of re-fabricating new structures is unavoidable to change the dimensions of metal-based metamaterial absorbers and further tune the wavelength of the absorption peak. Hence, it is still urgently required to introduce the suitable material into metamaterial absorbers to achieve the active tunability, such as the continuous tuning of the absorption frequency.

Recently, graphene^24^,^25^, the first truly two-dimensional material to be observed and isolated in nature, is a single layer of carbon atoms gathered in a honeycomb lattice. Because of the unique electronic band structure that the energy-momentum relation for electrons is liner over a wide range of energies rather than quadratic^26^, graphene exhibits many exotic optical features including extreme field confinement, low damping losses, and advantageous tunability^27^,^28^,^29^. Additionally, the well-known surface plasmon polaritons (SPPs), sub-wavelength localized electromagnetic waves, are supported by the graphene in the mid-infrared region^30^,^31^,^32^,^33^. More importantly, graphene owns the original gate-voltage-dependent optical feature^34^,^35^ that the chemical potential of graphene can be changed by means of external gate voltages. Graphene-based SPP waves hence can be engineered conveniently by using external gate voltages to vary the surface conductivity of graphene in real-time^36^, without changing structural dimensions of devices, because the surface conductivity of graphene depends on the chemical potential. This gate-voltage method is preferable for realizing the tunability instead of directly re-fabricating a new structure, as would be needed for metallic devices. Therefore, graphene is emerging as a possible platform for new-generation electrically controlled nano-plasmonic devices. The introduction of graphene will opens up an opportunity to obtain active frequency tuning for metamaterial absorbers.

Currently, a great diversity of graphene-based metamaterials^37^,^38^,^39^ have been devised for enhancing optical absorption, such as, graphene discs^40^, ribbons^41^, rings^42^, even the monolayer graphene coated by metallic gratings^43^. Herein, we propose a wide-angle plasmonic narrowband perfect absorber based on the periodic double-layer graphene ribbon arrays separated from a metallic ground plate by an ultra-thick dielectric layer. The versatile CMT is applied to predict the perfect absorption response. The FDTD simulation is utilized to reveal the underlying physics and investigate comprehensively the proposed perfect absorber. Simulation results exhibits that the anti-symmetric dipole-dipole coupling resonance in double-layer graphene ribbons acts as the electrical resonance to suppress the reflection, due to the impedance matching. The ultra-thick metallic substrate is used for restricting the transmission. The incident electromagnetic energy is efficiently confined in the interlayer between graphene ribbons and the metallic plate. The outstanding perfect absorption peak with the FWHM of 300 nm is obtained. In addition to varying geometrical parameters of the absorber, a small change in the chemical potential of graphene can be used for controlling the spectral position of the absorption peak. Meanwhile, this device exhibits good absorption stability over a wide angle range of incidence around ± 60° at least. Such absorber will benefits the fabrication of nanophotonic devices and plays an important role in mid-infrared optical filtering and storage.

## Results

**Figure 1:**
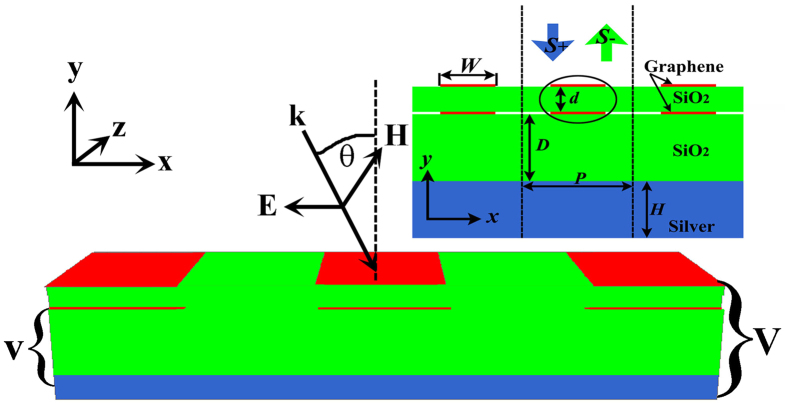
Schematic of the graphene-based perfect absorber and the incident light polarization configuration. The inset shows the *x*-*y* cross-section of the proposed absorber. The periodic double-layer graphene ribbons with the coupling distance *d*, width *W* and period *P* are constructed. The ribbon arrays are separated from an ultra-thick silver ground plate by a silica layer with thickness *D*. The thickness of the silver substrate is *H*.

First, the structure of the proposed absorber composed of the periodic double-layer graphene ribbon array placed near a metallic ground plate coated by a dielectric layer is depicted schematically in the Fig. 1. The *x*-*y* cross-section of this device with the corresponding geometrical description is shown in the inset. The double-layer graphene ribbons with identical width of *W* are separated by the silica layer with the thickness of *d*. The period of the ribbons array is *P*. The cavity between the metallic ground plate and the lower graphene layer is filled also by the silica layer with thickness of *D*. The thickness of the metallic ground plate is *H*. When the plane wave with the polarization along the *x* axis irradiates normally this structure, the second-order dipole resonance should form on the single graphene ribbon^44^. For the double-layer graphene ribbons with a small separation of *d* = 50 nm, the anti-symmetric plasmonic dipole-dipole coupling resonance^45^ should form based on the near-field coupling. The transmission from the whole structure is very close to zero (*T* = 0), as the thickness of the metallic ground plate with *H* = 800 nm is much larger than the penetration depth of electromagnetic waves. When the double-layer graphene ribbons are considered as a whole and called the anti-symmetric dipole-dipole coupling resonator, the proposed device behaves as such construction of the single port coupled with a resonator. The versatile CMT^46^,^47^,^48^ can illustrate very adequately the property of this configuration.

As shown in the inset of Fig. 1, the amplitudes of the normalized incoming and outgoing waves transmitted through the port are depicted as *S*_+_, and *S*_−_, respectively. The resonant frequency of the anti-symmetric dipole-dipole coupling mode formed between double-layer graphene ribbons is *ω*_0_. The dissipative and radiative losses occurring in the anti-symmetric coupling resonator are represented by *τ*_*a*_^−1^ and *τ*_*r*_^−1^, respectively. The coupling strength between the incident wave and the anti-symmetric resonator is characterized by *τ*_*c*_^−1^. When the incident wave with the frequency *ω* is launched into this system, the time-evolution-normalized amplitude of the anti-symmetric dipole-dipole coupling resonator is expressed as


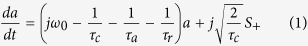


Thanks to the power conservation and time reversal symmetry, the relationship between the amplitudes of the normalized incoming and outgoing waves transmitted through the only port is described as


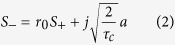


Here, the *r*_0_ is the reflectance of this system without the double-layer graphene ribbon array. Because the metallic ground plate behaves as a mirror, all incident waves will be reflected directly at the upper surface of the metallic plate. Therefore, we can obtain *r*_0_ = −1. *j* stands for the imaginary unit. Isolating the *S*_−_/*S*_+_ and going to the frequency domain *e*^+ *jωt*^, the reflectance of the device can be obtained and expressed as


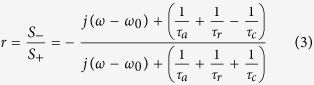


The impedance of this system can be defined as 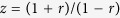
15. As this system owns only one anti-symmetric coupling resonator formed between double-layer graphene ribbons, the Lorentzian-shaped resonance should have the minimum at *ω* = *ω*_0_ for the reflection spectrum. At the *ω* = *ω*_0,_ the effective impedance of this system is 
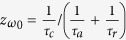
. If the effective impedance of this system matches to that of the free space (*z*_0_ = 1), the radiative losses can be neglected, corresponding to *τ*_*r*_^−1^ ≈ 0. Hence, only the condition of *τ*_*a*_^−1^ = *τ*_*c*_^−1^ should be satisfied. At the *ω* = *ω*_0_, the impedance matching is realized and *r* = 0. Thus, the absorption is *A* = 1 − *T* − |*r*|^2^ = 1. In other word, the perfect absorption response will be achieved at the *ω* = *ω*_0_.

The reflection of this system is


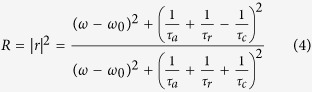


The absorption of this structure can be given by


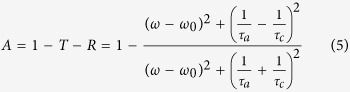


**Figure 2:**
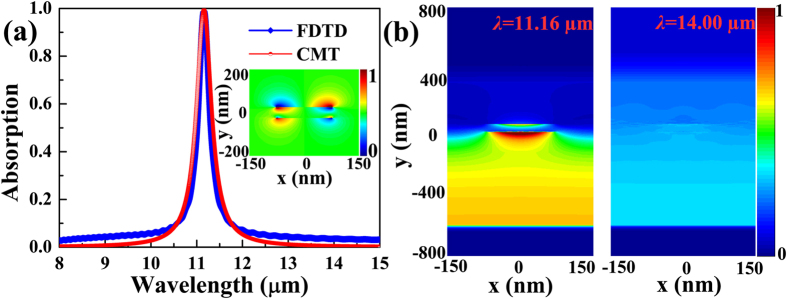
Absorption spectra and electromagnetic field distributions of the graphene-based device. (**a**) Absorption spectra obtained by the CMT and FDTD simulation. The inset illustrates the electric field (*E*_*y*_) distributions of the unit-cell double-layer graphene ribbons at the resonant wavelength of *λ*_0_ = 11.16 μm. (**b**) Magnetic field (*H*_*z*_) distributions of the entire unit cell at wavelengths of *λ*_0_ = 11.16 μm and *λ* = 14.00 μm, respectively.

The CMT predicted absorption spectrum is shown in Fig. 2(a), and marked by the red line. Here, *τ*_*a*_^−1^ = *τ*_*c*_^−1^ = 1*10^12 ^Hz are considered for obtaining a useful impedance matching. The resonant wavelength *λ*_0_ = 2π*c*/*ω*_0_ is assumed as 11.16 μm, here *c* is the velocity of light in vacuum. Obviously, the perfect absorption feature occurs. In order to confirm the CMT-predicted perfect absorption response and further investigate comprehensively the proposed absorber, the numerical simulation is performed by the Lumerical FDTD Solutions.

In the numerical calculations, the graphene ribbon is modeled as an ultra-thin film with a thickness of Δ (in the *y* direction). The surface conductivity (*σ*_*g*_) of the graphene is governed by the Kubo formula^49^,^50^ that depends on the momentum relaxation time *τ*, temperature *T*, chemical potential *μ*_*c*_, and incident wavelength *λ* (frequency *ω*). At the room temperature and for the highly doped graphene in the simulated mid-infrared spectral range, the chemical potential is always above half of the photon energy. The Kubo equation hence is reduced to^51^ 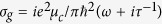, where the intraband transition dominates and the interband transition is neglected. The equivalent permittivity of graphene is calculated by the equation^52^: 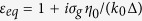, where 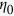
(≈377 Ω) is the intrinsic impedance of vacuum, and 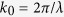. The thickness of the monolayer graphene is assumed to be Δ = 1 nm which is reasonable, although its real thickness is 0.34 nm. Because the equivalent permittivity *ε*_*eq*_ of graphene is thickness-dependent^52^, the different values of the Δ will give rise to the corresponding equivalent permittivity and these extremely small values of the thickness can lead to similar simulation results. The chemical potential of graphene is assumed first to be *μ*_*c*_ = 0.4 eV, and the momentum relaxation time is chosen as *τ* = 0.5 ps. The metal is chosen as the silver with the dispersive permittivity characterized by the Drude model^53^,^54^,^55^ 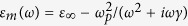. Here, *ε*_*∞*_ = 3.7 is the dielectric constant at infinite angular frequency; *ω*_*p*_ = 9.1 eV is the bulk plasma frequency representing the natural frequency of the oscillations of free conduction electrons; *γ* = 0.018 eV stands for the damping frequency of the oscillations. In the considered mid-infrared region, the silica is assumed to be a non-dispersive dielectric with the index of 1.45. The unit cell with *P* = 300 nm, *W* = 150 nm, and *D* = 600 nm is performed. Periodic boundary conditions are applied along the *y* directions and perfectly matched layers are employed in the *x* direction. A plane wave with the electric field parallel to the *x* axis illuminates normally the periodic structure. We use the non-uniform mesh, and the minimum mesh size inside the graphene layer equals 0.1 nm and gradually increases outside the graphene sheet, for saving storage space and computing time. 2D-FDTD simulated results are presented in Fig. 2.

In the Fig. 2(a), the simulated absorption spectrum indicated by the blue curve is shown and the perfect absorption peak with the resonant wavelength of *λ*_0_ = 11.16 μm appears distinctly. The FWHM of the absorption peak is 300 nm. Simulated results indeed confirm that the perfect narrowband absorber can be achieved by the proposed graphene-based structure. The simulated phenomenon is agreement with that predicted by the CMT. The electric field *E*_*y*_ distributions of the unit-cell double-layer graphene ribbons at the resonant wavelength of *λ*_0_ = 11.16 μm are displayed in the inset. Obviously, the second-order dipole resonance is supported by the single graphene ribbon. When the double-layer graphene ribbons are considered as a whole, the anti-symmetric plasmonic dipole-dipole binding indeed forms based on the near-field coupling. The entire structure of graphene ribbons hence acts as an anti-symmetric dipole-dipole coupling plasmonic resonator. The electrical resonance hence occurs at the graphene ribbons and the incident electromagnetic energy is confined and introduced into this system. To better understand the underlying physics of the perfect absorption response, the magnetic field (*H*_*z*_) distributions of the entire unit cell at wavelengths of *λ*_0_ = 11.16 μm and *λ* = 14.00 μm, are shown in Fig. 2(b). For the resonant wavelength of *λ*_0_ = 11.16 μm, the electrical resonance yields at the anti-symmetric coupling plasmonic resonator formed by the double-layer graphene ribbons. The effective impedance of the vertically coupling graphene ribbons matches to that of the free space, and the reflection thus is suppressed. The transmission from this system is also inhibited due to the ultra-thick metallic ground plate. All incident electromagnetic energy is efficiently confined in the silica interlayer between the graphene ribbons and the metallic ground plate. Therefore, the perfect absorption feature is achieved by the simple graphene-based structure. On the contrary, the electrical resonance of the double-layer graphene ribbons and the effective impedance matching condition cannot be formed at the other nor-resonant wavelengths. The most incident power is reflected and cannot be confined by this device, as shown in Fig. 2(b) at the incident wavelength of *λ* = 14.00 μm.

**Figure 3:**
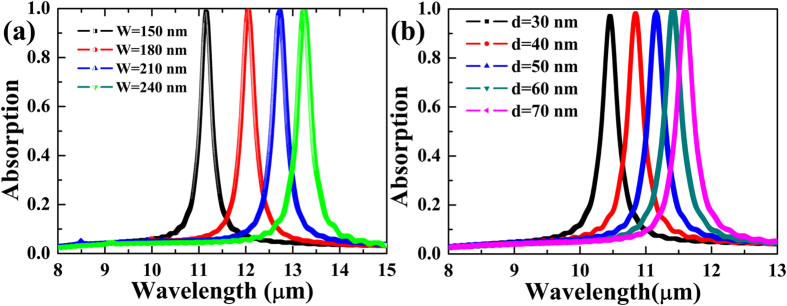
Absorption spectra for the geometric parameters of double-layer graphene ribbons. Absorption spectra with different ribbon widths *W* (**a**) and vertically coupling distances *d* between ribbons (**b**).

Now that the spectral position of the perfect absorption peak corresponds to the resonant wavelength of the anti-symmetric coupling dipole-dipole resonator formed on the double-layer ribbons, the change in the resonant wavelength of the anti-symmetric coupling resonator can be utilized to tune the spectral position of the absorption peak. For anti-symmetric coupling dipole-dipole resonator, the changes in the geometrical parameters of double-layer graphene ribbons should have a direct effect on its resonant wavelength. Thus, the changes in the geometrical parameters of graphene ribbons can be used first for tune the spectral position of the absorption peak *d* = 50 nm and the duty ratio of *W*/*P* = 0.5 are fixed. Other structural and material parameters are unchanged. The structure with the simultaneous change in the width *W* of double-layer ribbons is simulated and results are presented in Fig. 3(a). It is well known that the wavelength of dipole resonance in the single graphene ribbon increases as the ribbon width increases^56^. So, the absorption peak tends to exhibit a red shift with the ribbon width increase, as shown in Fig. 3(a) where the absorption spectra with ribbon widths of *W* = 150, 180, 210, and 240 nm are offered. The obvious width-dependent tunability is achieved. In addition, the real part of the wave vector of the anti-symmetric dipole-dipole coupling resonance increases with the vertical coupling distance between ribbons increase^45^. The resonant wavelength of the absorption peak hence should show a red shift as the coupling distance *d* increases. Simulated absorption spectra with different coupling distances are displayed in Fig. 3(b) where the other simulated parameters are same as that used in Fig. 2(b). The coupling distance varies from 30 to 70 nm, and the resonant wavelength of the absorption peak increases from 10.45 to 11.61 μm. The coupling-distance-dependent tunability is also confirmed.

**Figure 4:**
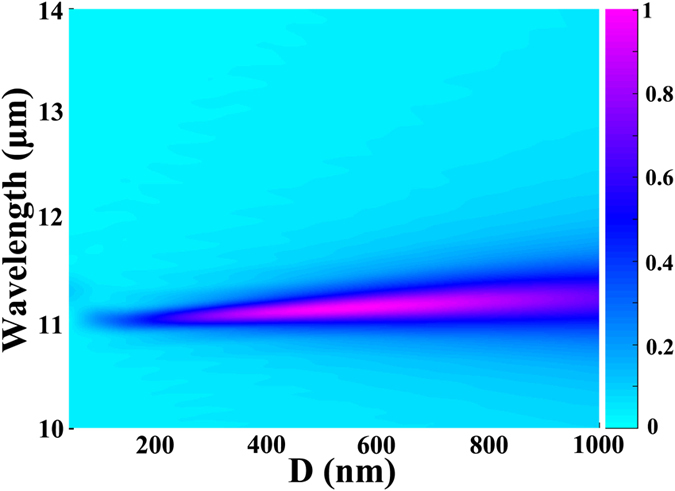
Absorption spectra under different distances (*D*). Simulated absorption spectra with different thicknesses (*D*) of the silica layer coated on the metallic ground plate. The thickness *D* varies from 50 to 1000 nm.

In addition, for the absorption peak, the most incident electromagnetic power is confined in the space between the lower graphene ribbon and the metallic ground plate. Hence, the height of the space should have a direct effect on the performance of this absorber. Numerical absorption spectra under different distances *D* are presented in Fig. 4. Simulated parameters are same to be that used for Fig. 2. According to this color map, it is found that the change in the distance *D* has little effect on the central wavelength of the absorption peak, whereas influences greatly the absorption and FWHM of the resonant peak. In other word, the absorption spectrum can be modulated by changing the thickness *D*, without changing the spectral position of the absorption peak. This phenomenon offered by the Fig. 4 can also be well understood. The effective impedance of the period double-layer graphene ribbons depends on the thickness *D* of the silica space^41^. As the thickness *D* increases from 50 to 600 nm, the perfect impedance matching to that of the free space is formed gradually. With the thickness *D* increase further, the perfect impedance matching is broken. Hence, the perfect absorber is achieved first and then is hampered with the increase of the thickness *D*. On the other hand, the anti-symmetric dipole-dipole coupling plasmonic resonance in double-layer graphene ribbons is localized around the graphene ribbons with deep sub-wavelength nano-scales, which are less than 50 nm as shown in the inset of Fig. 2(a). The change in the thickness of the dielectric layer from 50 to 1000 nm hence has no effect on the resonant wavelength of the anti-symmetric dipole-dipole coupling resonator. The spectral position of the absorption peak is kept unchanged as the thickness *D* increases from 50 to 1000 nm.

**Figure 5:**
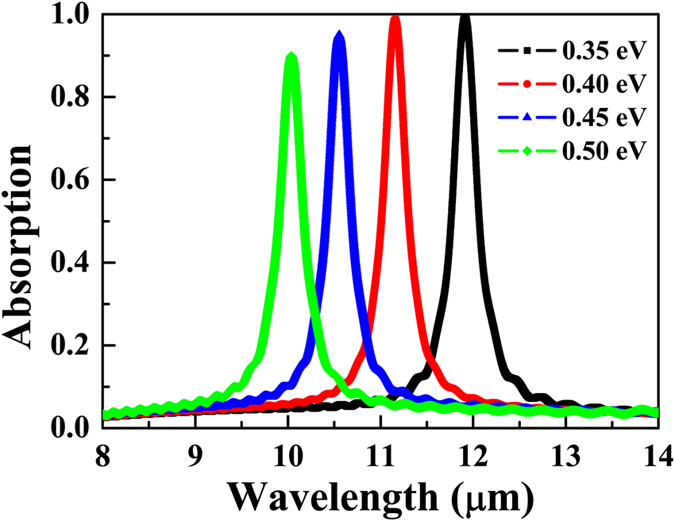
Absorption spectra for different chemical potentials (*μ*_*c*_) of graphene ribbons. Numerical absorption spectra with the chemical potential of double-layer graphene ribbons varying from 0.35 to 0.5 eV with an increment of 0.05 eV.

Besides the geometrical tunability, the graphene-based absorber is particular to own the novel gate-voltage-dependent feature that the chemical potential of graphene can be dynamically tuned by applying external gate voltages, as shown in Fig. 1 where the two external gates may be used for controlling the chemical potential. Without re-fabricating a new structure, only a small change in the chemical potential of graphene can vary the plasmonic resonant wavelength because the surface conductivity of graphene depends directly on the chemical potential. Here, the effect of the change in chemical potentials of double-layer graphene ribbons on the spectral position of the absorption peak is calculated numerically. Simulated absorption spectra with chemical potentials of *μ*_*c*_ = 0.35, 0.4, 0.45, and 0.5 eV, respectively, are shown in Fig. 5. Other simulation parameters are same as that in Fig. 2(b). A comparison of four curves indicates that the resonant wavelength of absorption peak shifts to a shorter wavelength with an increase of the chemical potential. It is mainly because that the real part of the wave vector of the anti-symmetric dipole-dipole coupling resonance decreases as the chemical potential increases^56^. In addition, it is clear that the absorption at the resonant peak decreases gradually as the chemical potential increases. It owes to that the impedance matching is broken when the chemical potential increases. The reflection cannot be suppressed completely. However, in the considered range of the chemical potential, the absorption is always larger than 90%. Hence, the chemical-potential-dependent tunability is obtained.

**Figure 6:**
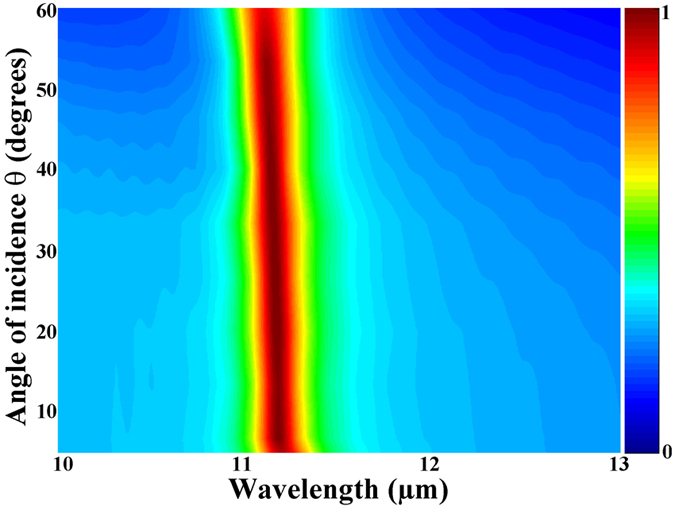
Absorption as a function of wavelength and the angle of incidence (*θ*). Simulated absorption spectra with different angles of incidence (*θ*). The angle varies from 5 to 60°.

It should be pointed out that the electromagnetic waves irradiate usually this absorber with an oblique incident angle in the practical applications. Thus, it is necessary to investigate the robustness of the proposed perfect absorber under the oblique incidence. Other simulation parameters are similar to that in Fig. 2(b). Further simulations with nor-normal incident angles are performed, and the absorption as a function of wavelength and the angle of incidence is displayed in Fig. 6. Based on this color map, it is obvious that the spectral position of the absorption peak nearly keeps unchanged over a wide angle range of incidence around ± 60°. At the same time, the narrowband absorption (great than 85%) is not limited to the normal incident angle but extended to at least 60°. Hence, the designed narrowband absorber operates well over a wide range of the angle of incidence, and the wide-angle graphene-based absorber is obtained.

## Discussion

To sum up, the periodic double-layer graphene ribbon arrays placed on a metallic ground plate coated by a dielectric space are proposed and investigated both numerically and theoretically. The well-known CMT is used for analyzing the structural property and predicting the perfect absorption feature. FDTD numerical simulation is utilized to give the underlying physics. Simulated results exhibit that the localized anti-symmetric dipole-dipole coupling modes occurs at double-layer graphene ribbons and acts as the electrical resonance to suppress the reflection, thanks to the impedance matching. The ultra-thick metallic ground plate is used for restricting the transmission from this system. The incident electromagnetic energy is efficiently confined in the interlayer between graphene ribbons and the metallic substrate, and the novel perfect absorber hence is achieved. The spectral position of the absorption peak can be dynamically tuned by a small change in the chemical potential, in addition to varying geometrical parameters of the absorber. Meanwhile, this device exhibits good absorption stability over a wide angle range of incidence around ± 60° at least. Such simple plasmonic perfect absorber may benefit the fabrication of nanophotonic devices for optical filtering and storage in the mid-infrared region.

## Methods

### Numerical simulations

The 2D finite-difference time-domain (FDTD) method is used for the numerical simulation and the commercial software of Lumerical FDTD Solutions is performed. Periodic boundary conditions are applied along the *y* directions and perfectly matched layers are employed in the *x* direction. A plane wave with the electric field parallel to the *x* axis illuminates normally the periodic structure. The non-uniform mesh is adopted, and the minimum mesh size inside the graphene layer equals 0.1 nm and gradually increases outside the graphene sheet, for saving storage space and computing time.

## Additional Information

**How to cite this article**: Li, H. *et al*. Tunable graphene-based mid-infrared plasmonic wide-angle narrowband perfect absorber. *Sci. Rep.* **6**, 36651; doi: 10.1038/srep36651 (2016).

**Publisher’s note**: Springer Nature remains neutral with regard to jurisdictional claims in published maps and institutional affiliations.
